# Effects of 2-bromoterguride, a dopamine D_2_ receptor partial agonist, on cognitive dysfunction and social aversion in rats

**DOI:** 10.1007/s00213-017-4747-x

**Published:** 2017-10-03

**Authors:** Emilia Tarland, Robert T. Franke, Heidrun Fink, Heinz H. Pertz, Jan Brosda

**Affiliations:** 1 Institute of Pharmacology and Toxicology, School of Veterinary Medicine, Freie Universität Berlin, 14195 Berlin, Germany; 2 Institute of Pharmacy, Freie Universität Berlin, 14195 Berlin, Germany; 3 Present Address: Bundesamt für Risikobewertung (BfR), Max-Dohrn-Str. 8-10, 10589 Berlin, Germany

**Keywords:** Prepulse inhibition, Cognitive deficit symptoms of schizophrenia, Novel object recognition, Social interaction, Rat, Dopamine D_2_ receptor partial agonist, Antipsychotic

## Abstract

**Rationale:**

2-Bromoterguride, a dopamine D_2_ receptor partial agonist with antagonist properties at serotonin 5-HT_2A_ receptors and α_2C_-adrenoceptors, meets the prerequisites of a putative atypical antipsychotic drug (APD). We recently showed that 2-bromoterguride is effective in tests of positive symptoms of schizophrenia in rats without inducing extrapyramidal side effects or metabolic changes.

**Objective:**

In continuation of our recent work, we now investigated the effect of 2-bromoterguride on apomorphine and phencyclidine (PCP)-induced disruptions of prepulse inhibition (PPI) of the acoustic startle response, a measure of sensory gating. In addition, we used subchronic PCP treatment to produce cognitive deficits and social aversion, and assessed the effect of 2-bromoterguride on the performance in the novel object recognition (NOR) task (model for studying cognitive deficit symptoms of schizophrenia) and the social interaction test (model for studying negative symptoms of schizophrenia). Finally, we extended the side effect profile of 2-bromoterguride by measuring the prolactin response to systemic administration of the drug in rats.

**Results:**

Treatment with 2-bromoterguride (0.1 and 0.3 mg/kg) reversed PPI deficits induced by apomorphine and PCP, respectively. Subchronic PCP induced impairments in object memory and social interaction behavior which were ameliorated by 2-bromoterguride but not by clozapine and aripiprazole, respectively. Prolactin concentration in blood serum was not elevated at 1, 2, or 4 h post-2-bromoterguride treatment, which further supports the safe and effective use of this drug.

**Conclusions:**

Our data support 2-bromoterguride as a promising APD candidate due to its beneficial effect on cognitive impairments and negative symptoms of schizophrenia.

## Introduction

The treatment of schizophrenia ideally involves reduction of positive symptoms, negative symptoms, and cognitive deficits. Positive symptoms such as hallucinations and delusions can be treated more or less satisfactorily with currently available antipsychotic drugs (APDs). However, negative symptoms (affective flattening, alogia, avolition, and anhedonia) and cognitive impairment often fail to respond to typical (first generation) APDs (Dunlop and Brandon 2015; Vreeker et al. 2015).

Emerging evidence from preclinical and clinical studies using atypical (second generation) APDs with additional affinities for multiple serotonin (5-HT) receptors, predominantly the 5-HT_2A_ subtype, provided renewed optimism for the pharmacological treatment of schizophrenia and other psychotic disorders (Aznar and Hervig 2016). Atypical APDs produce less extrapyramidal side effects (EPS), tardive dyskinesia, and hyperprolactinemia than typical APDs but their antagonistic effects at histamine H_1_ and 5-HT_2C_ receptors might induce insulin resistance, weight gain, diabetes, and other secondary conditions (Meltzer 2013; Kim et al. 2007; Ücok and Gaebel 2008). Additionally, and most importantly, atypical APDs are apparently no more effective than typical APDs regarding negative and cognitive symptoms of schizophrenia (Leucht et al. 2013).

**Fig. 1:**
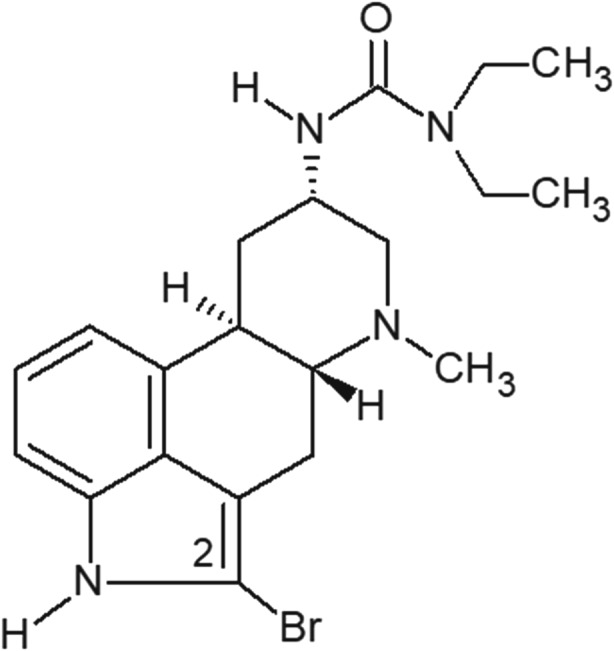
Chemical structure of 2-bromoterguride

Dopamine D_2_ receptor partial agonists such as aripiprazole represent the latest advancement in the treatment of schizophrenia. The intrinsic dopamine-stabilizing effect of partial agonists can adjust the levels of dopamine through decreased postsynaptic transmission in the mesolimbic system when dopamine is elevated, and through increased transmission in the mesocortical system when the dopamine level is low (de Bartolomeis et al. 2015). 2-Bromoterguride (Fig. 1) is a dopamine D_2_ receptor partial agonist that mechanistically resembles aripiprazole. 2-Bromoterguride is the dihydro derivative of the dopamine antagonist bromerguride (2-bromolisuride), a compound that behaved as an atypical APD (Löschmann et al. 1992). Interestingly, 2-bromoterguride possesses a higher affinity for 5-HT_2A_ receptors and α_2C_-adrenoceptors, and a lower affinity for histamine H_1_ receptors than aripiprazole (Jantschak et al. 2013). We recently demonstrated that 2-bromoterguride inhibits amphetamine-induced locomotion (AIL) and conditioned avoidance response (CAR) in rats, suggesting antipsychotic action. Furthermore, neither acute nor chronic treatment with 2-bromoterguride induced catalepsy or altered body fat composition and body weight in rats (Franke et al. 2016; Jantschak et al. 2013).

To investigate the prospective effects of 2-bromoterguride as a clozapine-like atypical APD, we induced disruptions of prepulse inhibition (PPI) of the acoustic startle reflex using the mixed dopamine D_1_/D_2_ receptor agonist apomorphine and the noncompetitive NMDA receptor antagonist phencyclidine (PCP). PPI is a pre-attentive process regulated by multiple neurotransmitter systems including dopaminergic, serotonergic, cholinergic, GABAergic, and glutamatergic systems (Geyer 1998). Apomorphine induces loss of PPI in a robust manner when administered to rats, and its effect can be blocked by typical APDs like haloperidol (Mansbach et al. 1988). In contrast, loss of PPI induced by PCP is insensitive to either dopaminergic or serotonergic antagonists but can be attenuated by selective α_2C_-adrenoceptor antagonists such as JP-1302 or atypical APDs such as clozapine and quetiapine (Sallinen et al. 2007; Swerdlow et al. 1996; Bakshi et al. 1994).

In contrast to acute PCP administration, subchronic PCP treatment triggers prefrontal cortical dopaminergic hypoactivity and a hyper-responsive state in the mesolimbic dopamine system, resembling the pathophysiology of schizophrenia (Jentsch et al. 1998). Behaviorally, subchronic PCP administration induces memory and learning deficits (for reviews, see Jentsch and Roth 1999; Meltzer et al. 2013; Neill et al. 2010). Atypical APDs in contrast to typical ones successfully attenuate the object memory-disrupting effects of PCP in rodents (Grayson et al. 2007; Horiguchi et al. 2012; Oyamada et al. 2015, Snigda et al. 2011). To study the effect of 2-bromoterguride on these cognitive disruptions, we used the one-trial object recognition test, commonly known as the novel object recognition task (NOR). NOR is a well-established model for assessment of visual learning and recognition memory in rodents (Ennaceur and Delacour 1988; Ennaceur 2010) and to evaluate the general efficacy of novel APDs to alleviate cognitive deficits (Grayson et al. 2015). In addition to its disrupting effect in rodent object memory, subchronic PCP induces social interaction impairments, which can be attenuated by compounds with combined D_2_/5-HT_2A_ antagonist and D_1_/5-HT_1A_ agonist properties but not by dopamine D_2_ selective agents (Bruins Slot et al. 2005; Neill et al. 2014; Snigdha and Neill 2008a,b). To assess the effect of 2-bromoterguride on PCP-induced social aversion, we used the social interaction test, an established paradigm to demonstrate the effect of putative therapies on the negative symptoms of schizophrenia (for review, see Wilson and Koenig 2014).

Finally, we wanted to know whether 2-bromoterguride is a prolactin-elevating APD. Elevated levels of serum prolactin may induce sexual dysfunction worsening negative schizophrenic symptoms (Leucht et al. 2013).

## Materials and methods

### Animals

Naïve male Sprague-Dawley rats (Élevage Janvier, Le Genest Isle, France) aged 10 weeks and with a mean weight of 450 g by the beginning of the experiments were used for the PPI (*n* = 98), NOR (*n* = 70) and social interaction (*n* = 98) experiments. Rats from the PPI study were additionally used for prolactin determination (*n* = 74). At this point of the study, the aim was to investigate the overall effectiveness of 2-bromoterguride on cognition and sociality in vivo. Therefore, and for reasons of comparability to data of our previous study (Franke et al. 2016), only one gender (males) was included to reduce the total number of animals. The animals were housed in groups of 3–4 per cage (type open-top IV polycarbonate cages; Ehret, Emmendingen, Germany) under standard laboratory conditions (room temperature, 22 ± 2 °C; relative humidity 55 ± 10%) on a 12 h light-dark schedule (lights on at 6:00 am). All experiments were conducted during the light phase between 9:00 am and 2:00 pm. Water and laboratory chow (Ssniff, Soest, Germany) was freely available and the cages were enriched with metal tubes and paper tissues. The animals were allowed 1 week of acclimatization upon arrival and gently handled 5 min/day (during the second week) by the person performing the experiments. All experimental procedures were approved by the Berlin State Authority (“Landesamt für Gesundheit und Soziales”) and performed in compliance with the German Animal Protection Law and the EU Directive 2010/63/EU for animal experiments.

### Drugs

2-Bromoterguride (Alfarma sro, Cernosice, Czech Republic) was suspended in 15% Cremophor® EL (Sigma-Aldrich, Steinheim, Germany), phencyclidine hydrochloride (Sigma-Aldrich, Steinheim, Germany) and haloperidol (Janssen Pharmaceuticals, Beerse, Belgium) were dissolved in 0.9% saline. Apomorphine hydrochloride hemihydrate (Sigma-Aldrich, Steinheim, Germany) was dissolved in 0.2% ascorbic acid solution, aripiprazole (Toronto Research Chemicals, Toronto, Canada) in 30% *N,N*-dimethylformamide and blended in 0.5% acetic acid. Clozapine (Abcam Biochemicals, Cambridge, UK) was dissolved in 0.1 N HCl and adjusted to pH neutrality. Compound doses and the elicited effect on behavior were thoroughly evaluated in pilot studies prior to the PPI, NOR, and social interaction experiments. All drugs were freshly prepared on the day of injection and administered in a volume of 1 ml/kg body weight.

### Prepulse inhibition of the acoustic startle response (ASR)

We measured PPI of the acoustic startle response (ASR) using a two-unit SR-LAB startle response system (San Diego Instruments, San Diego, CA) placed in an experimental chamber with 43 dB ambient noise level. The startle response cabinets were sound isolated and contained an acrylic cylinder (non-restrictive, 9 cm in diameter) attached to a platform. The delivery of acoustic stimuli was operated by the SR-LAB interface system and emitted via loudspeakers above the cylinder. A piezoelectric accelerometer sensor installed beneath the platform transduced cylinder vibrations into analogue signals, which were digitized and stored by the SR-LAB software. A short test session was performed 2–4 days prior to the PPI experiment to distribute the rats into balanced treatment groups based on their mean baseline startle magnitudes. For the PPI experiment, the rats were pre-treated with 2-bromoterguride (0.1 or 0.3 mg/kg, i.p.), clozapine (5.0 mg/kg, i.p.), haloperidol (0.1 mg/kg, i.p.), or vehicle (saline 0.9%, i.p.) 30 min prior to the test, followed by either PCP (1.5 mg/kg, s.c.), apomorphine (0.5 mg/kg, s.c.), or vehicle (saline 0.9%, s.c.) 20 min later. The animals were placed into the startle chambers and the session initiated with a 5-min acclimatization period. Background noise of 70 dB was emitted during the complete session. A total of 100 trials were presented in a pseudorandom order with variable inter-trial intervals (7–23 s). The protocol consisted of 20 startle trials (pulse-alone, 118 dB sound pressure level (SPL), duration 40 ms), 10 prepulse trials (86 dB SPL, duration 20 ms), 10 no-stim trials, and 40 prepulse-pulse trials. Prepulse-pulse trials consisted of a single 118 dB pulse preceded by a 74-, 78-, 82-, or 86-dB prepulse (20 ms duration) emitted 120 ms before the pulse onset. In addition, 10 pulse-alone trials were carried out at the beginning (startle block 1) and the end of the session (startle block 2) to measure habituation to the startle stimulus. These trials were excluded from startle magnitude calculations. Data was measured during a 100-ms time window after stimulus onset and averaged for each animal and trial type. PPI of the ASR was calculated for prepulse-pulse trials as a percentage of pulse-alone startle magnitude [(mean startle magnitude for pulse-alone trials − mean startle magnitudes for prepulse-pulse trials)/mean startle magnitude for pulse-alone trials] × 100. Pilot experiments were conducted to define doses of apomorphine and PCP which induced a robust loss of PPI, and to examine the effect of 2-bromoterguride on startle activity and PPI alone.

### Novel object recognition

The rats were administered with PCP (5.0 mg/kg) or vehicle (0.9% saline) i.p. twice a day (at 8:00 am and 5:00 pm) for 7 days. After a 14-day drug wash-out period, the animals were habituated to a 50 × 50 × 32 cm-sized dark colored acrylic arena placed in a sound-isolated chamber with dimmed lightning (5 lux) over 3 days. The first habituation was performed in littermate groups for 15 min. On days two and three, the rats were placed alone in the arena for 10 min. On day four, the rats were injected with either 2-bromoterguride (0.1 or 0.3 mg/kg, i.p.), clozapine (5.0 mg/kg, i.p.), or vehicle (0.9% saline, 1 ml/kg, i.p.) 30 min prior to the experiment.

In the acquisition trial (3 min), each rat was exposed to two identical objects, placed in opposite diagonal corners of the arena (positioned 12 cm from the walls). Subsequently, the rat was placed in a holding cage for a 1-min inter-trial interval; meanwhile, both objects were replaced (novel object + triplicate of familiar object). Finally, during the retention trial, the rat was re-introduced to the arena and allowed to explore the objects for 3 min. We used bright-colored glass bottles with metal caps, 12 × 12 × 6 cm and dark colored glass bottles with a blue plastic wrap, 12 × 12 × 6 cm, respectively. Objects were counterbalanced as novel or familiar objects in the treatment groups. Object attribute sensitivity and animal preferences was thoroughly evaluated in pilot studies to ensure that the objects elicited the same level of spontaneous investigation. The position of the novel object was counterbalanced (left/right) to eliminate spatial bias. The arena (after the acquisition and retention trial) and objects (after each animal) were cleaned with a 1:3 mix of isopropyl alcohol (70%) and meliseptol® (B. Braun Melsungen AG, Germany) to remove olfactory traces. The experiments were video recorded and scored by a blinded experimenter. Object exploration was defined as sniffing, licking, biting, or touching the object from < 1 cm distance, but not climbing on the object. Animals which failed to explore one or both of the objects for less than 4 s during the acquisition or retention trial were excluded from the data analysis. Discrimination index was calculated from: [(time spent exploring the novel object − time spent exploring the familiar object)/total exploration time]. Track length was measured with the software Videomot2 (TSE-Systems, Berlin, Germany).

### Social interaction

Half of the rats were treated with PCP (5.0 mg/kg, i.p.) or vehicle (0.9% saline, i.p.) twice a day (at 8:00 am and 5:00 pm) for 7 days. The other half remained untreated, housed in an adjacent room, and were brought into the lab 30 min prior to experimental procedures. After a 14-day drug wash-out period, all animals were habituated to a 50 × 50 × 32 cm-sized arena as described for the NOR test. On the test day, PCP- or vehicle-treated rats received a dose of either 2-bromoterguride (0.1 or 0.3 mg/kg, i.p.), aripiprazole (3.0 mg/kg, i.p.), or vehicle (0.9% saline, i.p.) 30 min prior to the social interaction test. During the test, each PCP- or vehicle-treated animal was paired with a weight matched (to max 30 g difference) untreated animal, placed together in the arena for 10 min, and video recorded for subsequent behavior analysis (Videomot2; TSE-Systems, Berlin, Germany). An inanimate object (a 33-cl aluminum soda can) was placed in the arena to measure the preference for interacting with an unfamiliar animal opposed to an unfamiliar object. The following social and non-social behaviors were scored by a blinded experimenter: following (the subject rat moves behind the unfamiliar conspecific), sniffing (investigative sniffing the snout, body, or anogenital region of the unfamiliar animal), climbing (climbing over the back of the conspecific or pushing the head and/or forepart beneath the conspecific), avoiding (actively turning away when approached by the conspecific) and object exploration (sniffing the object from < 1 cm distance). An overall social behavior parameter was calculated as the sum of times engaged in the abovementioned social behaviors. To assess locomotion, line crossings were manually scored using a 9 × 9 squared grid. Treated rats were marked with dark stripes on the back to distinguish them from untreated rats during the video analysis.

### Prolactin

The rats received an injection of 2-bromoterguride (0.1 or 0.3 mg/kg, i.p.), haloperidol (0.5 mg/kg, i.p.), or vehicle (saline 0.9%, i.p.), and were sacrificed 1, 2, or 4 h later by decapitation. To avoid stress-related prolactin release, the rats were only handled by a familiar experimenter. Trunk blood was collected into standard 2 ml Eppendorf® tubes and left to clot for 40 min at room temperature. The samples were centrifuged 10 min at 21 °C with 4000 rpm, blood serum collected into aliquots and stored at −80 °C until prolactin determination. Enzyme-linked immunosorbent assay (ELISA) was performed to assess prolactin levels using a commercial available rat prolactin ELISA kit following the instructions of the kit manufacturer (DRG Instruments, Marburg, Germany). Samples were analyzed in duplicates in the same assay (MTPL-Reader “E-LizaMat” 3000, DRG Instruments, Marburg, Germany) and two rat prolactin control samples (DRG Instruments, Marburg, Germany) containing a mean of 15.8 ng/μl respective 29.6 ng/μl prolactin were used for internal quality verification. The assay sensitivity was 0.6 ng/ml and the intra- and inter-variability coefficients were 3.7 and 10.4%, respectively.

### Data presentation and analysis

Statistical analysis was performed with SigmaPlot 11 (Systat Software, Erkrath, Germany). Two-way repeated measures (RM) analysis of variance (ANOVA) with treatment as between-subjects factor and prepulse intensity as within-subjects factor was conducted to determine whether pretreatment with 2-bromoterguride, haloperidol, or clozapine reversed the effects of apomorphine or PCP on PPI. Startle habituation data were analyzed with two-way repeated RM ANOVA with treatment as between-subjects factor and startle block as within-subjects factor. Mean startle magnitude data for pulse-alone trials, for prepulse-elicited reactivity, and reactivity on no-stim trials were analyzed with one-way ANOVA. NOR and social interaction data were analyzed by one-way or two-way ANOVAs according to the parameter and experimental design. Prolactin quantity data were analyzed by two-way ANOVA with treatment and time as between-subjects factors. Post hoc pairwise comparisons (Holm-Sidak method) were performed when appropriate. *P* values < 0.05 were considered significant and all data presented as mean ± standard error of the mean (SEM).

## Results

### Prepulse inhibition of the acoustic startle response

**Fig. 2:**
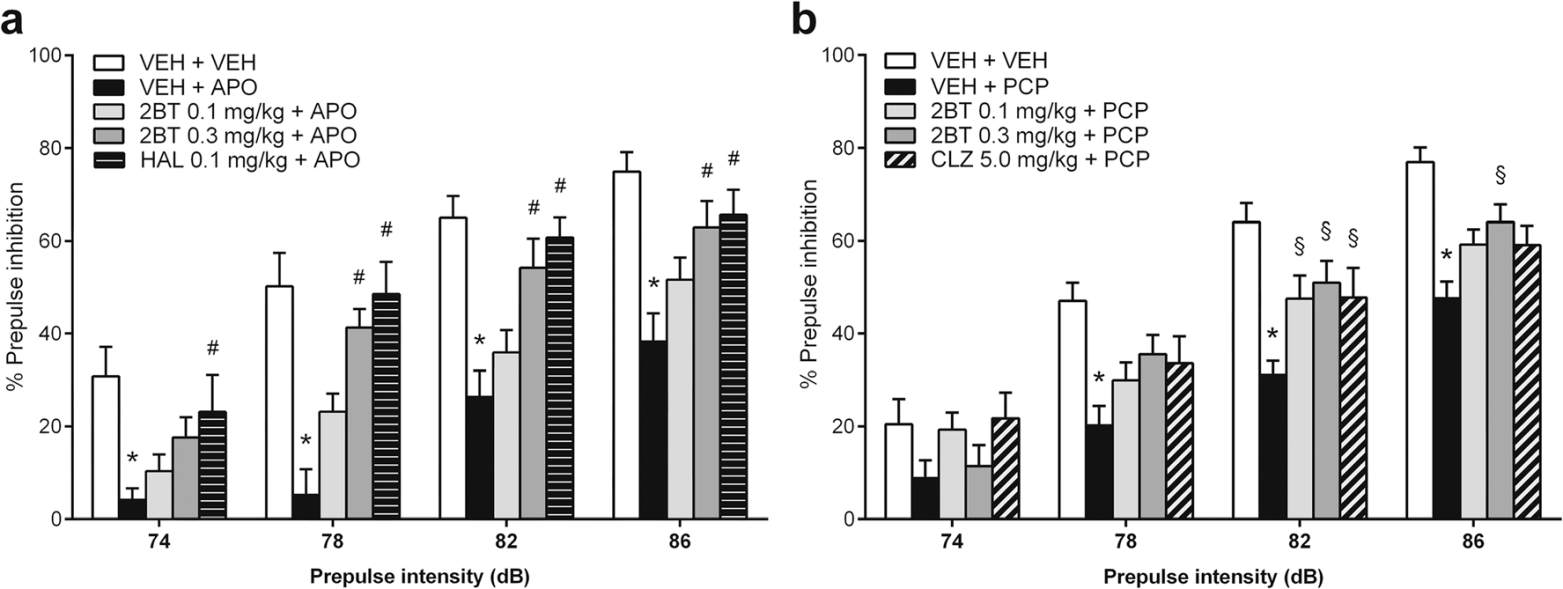
Effects of **a** 2-bromoterguride (0.1 and 0.3 mg/kg) and haloperidol (0.1 mg/kg) on acute apomorphine (0.5 mg/kg)-induced prepulse inhibition (PPI) deficits, and **b** 2-bromoterguride (0.1 and 0.3 mg/kg) and clozapine (5.0 mg/kg) on acute phencyclidine (1.5 mg/kg)-induced PPI deficits in male rats. Data are expressed as mean + SEM of *n* = 9–10 rats per group. ^*^ *P <* 0.05 versus controls (VEH + VEH); ^#^ *P <* 0.05 versus apomorphine (VEH + APO); ^§^ *P <* 0.05 versus phencyclidine. 2BT: 2-bromoterguride; APO: apomorphine; CLZ: clozapine; HAL: haloperidol; PCP: phencyclidine; VEH: vehicle

Acute apomorphine administration induced a robust loss of PPI compared to controls, which was attenuated by 2-bromoterguride or haloperidol treatment. We observed significant main effects for the factors treatment (*F*_(4,132)_ = 12.7, *P <* 0.001) and prepulse intensity (*F*_(3,132)_ = 113.9, *P <* 0.001). 2-Bromoterguride (0.3 mg/kg) prevented the apomorphine-induced PPI deficits at 78, 82, and 86 dB (*P <* 0.003 each), an effect that was elicited by haloperidol (0.1 mg/kg) at 74 (*P =* 0.044), 78, 82, and 86 dB (*P <* 0.002 each) (Fig. 2a). Further, 2-bromoterguride and clozapine attenuated PPI deficits induced by PCP. ANOVA revealed significant effects for the factors treatment (*F*_(4,132)_ = 6.4, *P <* 0.001), prepulse intensity (*F*_(3,132)_ = 207.8, *P <* 0.001), and the interaction of these factors (*F*_(12,132)_ = 2.2, *P =* 0.016). Post hoc comparisons revealed that both doses of 2-bromoterguride and clozapine ameliorated the PPI impairment at 82 dB (0.1 mg/kg: *P <* 0.007; 0.3 mg/kg: *P <* 0.009; clozapine: *P =* 0.017). Additionally, 0.3 mg/kg 2-bromoterguride was also effective with a prepulse of 86 dB (*P <* 0.034) (Fig. 2b). The ASR magnitude was affected by the factor treatment (*F*_(4,132)_ = 4.5, *P =* 0.004). Apomorphine alone (*P =* 0.013), or in combination with 0.1 mg/kg 2-bromoterguride (*P <* 0.027), increased the startle reaction (Table 1). All treatment groups habituated to the startle stimuli as illustrated by the mean startle reactivity in the first 10 pulse-alone trials (startle block 1) compared to the last 10 pulse-alone trials (startle block 2) (*P <* 0.001). We observed a main effect of startle block (*F*_(1,117)_ = 233.7, *P <* 0.001) and an interaction between treatment and startle block (*F*_(10,117)_ = 3.6, *P <* 0.001) (Table 1). Pilot experiments showed that (i) the used doses of apomorphine/PCP induced a PPI-disruptive effect and (ii) the used doses of 2-bromoterguride alone did not affect startle activity or PPI.

**Table 1:** Effects of apomorphine (0.5 mg/kg) and phencyclidine (1.5 mg/kg) in the absence or presence of 2-bromoterguride, haloperidol, and clozapine on startle habituation and startle reactivity

	Mean startle reactivity		
Treatment	Startle block 1 (first ten pulse-alone trials)	Startle block 2 (last ten pulse-alone trials)	Startle reaction (pulse-alone trials)
VEH + VEH VEH + APO 2BT (0.1 mg/kg) + APO 2BT (0.3 mg/kg) + APO HAL (0.1 mg/kg) + APO	4393 ± 568 7640 ± 540 8519 ± 736 5625 ± 925 5196 ± 1094	^#^2655 ± 817 ^#^4050 ± 682 ^#^3251 ± 600 ^#^3533 ± 576 ^#^3130 ± 622	2973 ± 486 ^*^6150 ± 621 ^*^5860 ± 657 4019 ± 633 4019 ± 883
VEH + VEH VEH + PCP 2BT (0.1 mg/kg) + PCP 2BT (0.3 mg/kg) + PCP CLZ (5.0 mg/kg) + PCP	5160 ± 737 6237 ± 732 5400 ± 795 5199 ± 443 5392 ± 806	^#^3430 ± 532 ^#^3284 ± 526 ^#^3387 ± 570 ^#^3635 ± 471 ^#^2972 ± 735	3684 ± 573 4425 ± 681 4211 ± 636 3715 ± 573 4278 ± 834

Data (in mV) are expressed as mean ± SEM

2BT 2-bromoterguride, APO apomorphine, CLZ clozapine, HAL haloperidol, PCP phencyclidine, VEH vehicle, (*n* = 9–10 rats per group)

^#^ *P <* 0.001 versus startle block 1; ^*^ *P <* 0.05 versus controls (VEH + VEH)

### Novel object recognition task

**Fig. 3:**
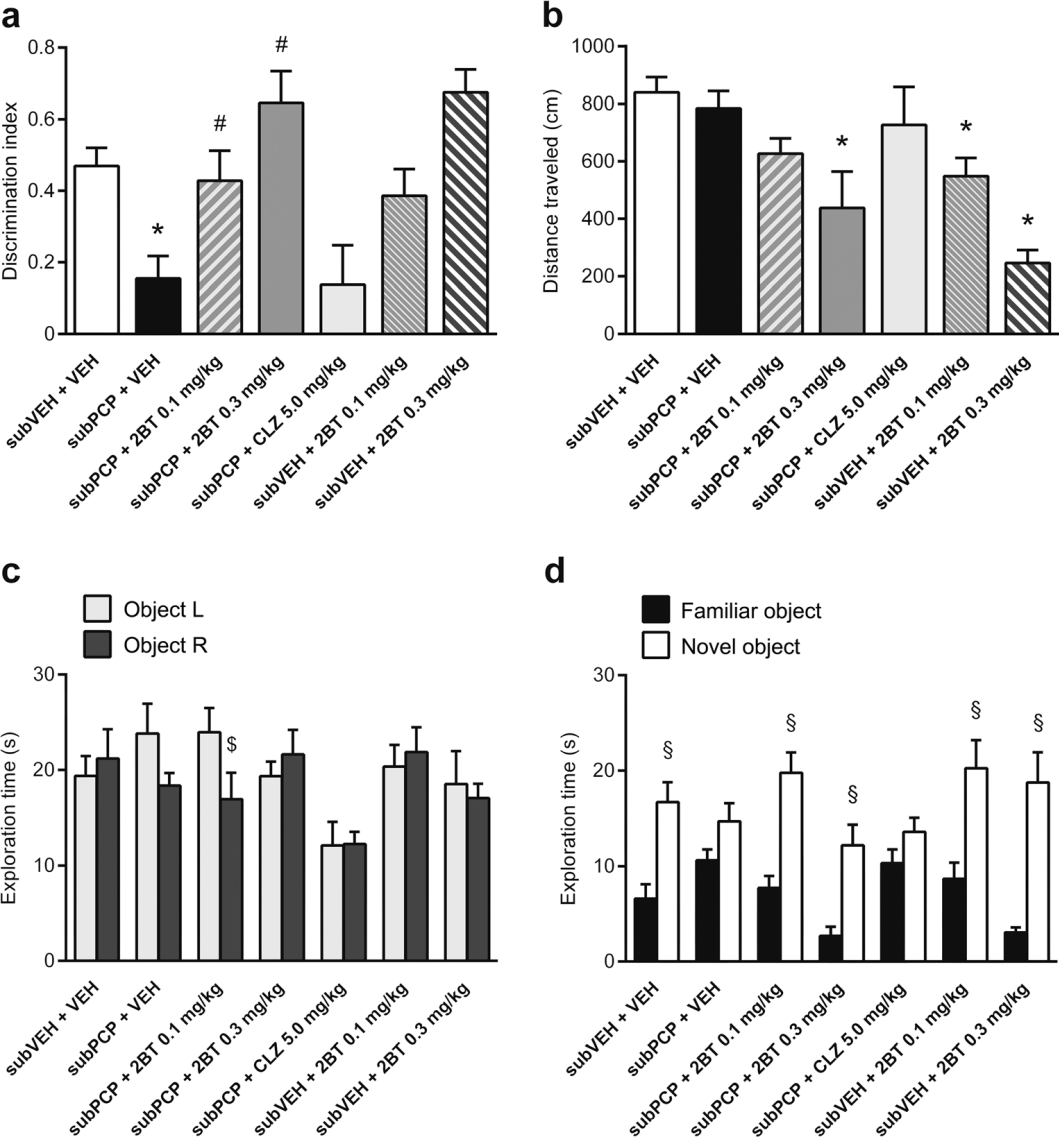
Effects of 2-bromoterguride (0.1 and 0.3 mg/kg) and clozapine (5.0 mg/kg) after subchronic phencyclidine (5.0 mg/kg) treatment, and 2-bromoterguride (0.1 and 0.3 mg/kg) alone on the **a** discrimination index, **b** distance traveled during the 3 min long retention trial, **c** exploration time of two identical objects (L and R) during the 3-min-long acquisition trial, and **d** exploration time of the familiar versus the novel object during the 3-min-long retention trial in the novel object recognition task (NOR) in male rats. Data are expressed as mean + SEM of *n* = 7–10 rats per group. ^*^ *P <* 0.05 versus controls (subVEH + VEH); ^#^ *P <* 0.05 versus phencyclidine (subPCP + VEH); ^$^ *P <* 0.05 versus the second identical object; ^§^ *P <* 0.05 versus familiar object. 2BT: 2-bromoterguride; CLZ: clozapine; PCP: phencyclidine; VEH: vehicle

2-Bromoterguride ameliorated subchronic PCP-induced cognitive impairment in the NOR task. A main effect of treatment on discrimination index was observed (*F*_(6,55)_ = 6.6, *P <* 0.001). Subchronic PCP led to a robust decrease in object recognition memory compared to controls (*P =* 0.027). 2-Bromoterguride resulted in levels similar to the control group (0.1 mg/kg: *P =* 0.44; 0.3 mg/kg: *P <* 0.001), an effect not elicited by clozapine. 2-Bromoterguride alone had no effect on NOR performance in control animals (Fig. 3a). Distance traveled during the retention trial was affected by treatment (*F*_(6,55)_ = 5.8, *P <* 0.001). Subchronic PCP in combination with 0.3 mg/kg 2-bromoterguride (*P =* 0.001) and 2-bromoterguride alone (0.1 mg/kg: *P =* 0.013; 0.3 mg/kg: *P <* 0.001) induced a reduction in track length compared to controls (Fig. 3b). However, 2-bromoterguride did not cause a decrease in object exploration time in the acquisition (Fig. 3c) or retention (Fig. 3d) trial. In the latter, ANOVA revealed significant main effects for the factor object (*F*_(1,55)_ = 122.2, *P* < 0.001) and the interaction of the factors object and treatment (*F*_(6,55)_ = 3.7, *P* = 0.004). Animals of all treatment groups favored the novel object over the familiar one (*P* < 0.001), with the exception of subchronic PCP in combination with vehicle and clozapine (Fig. 3d).

### Social interaction test

**Fig. 4:**
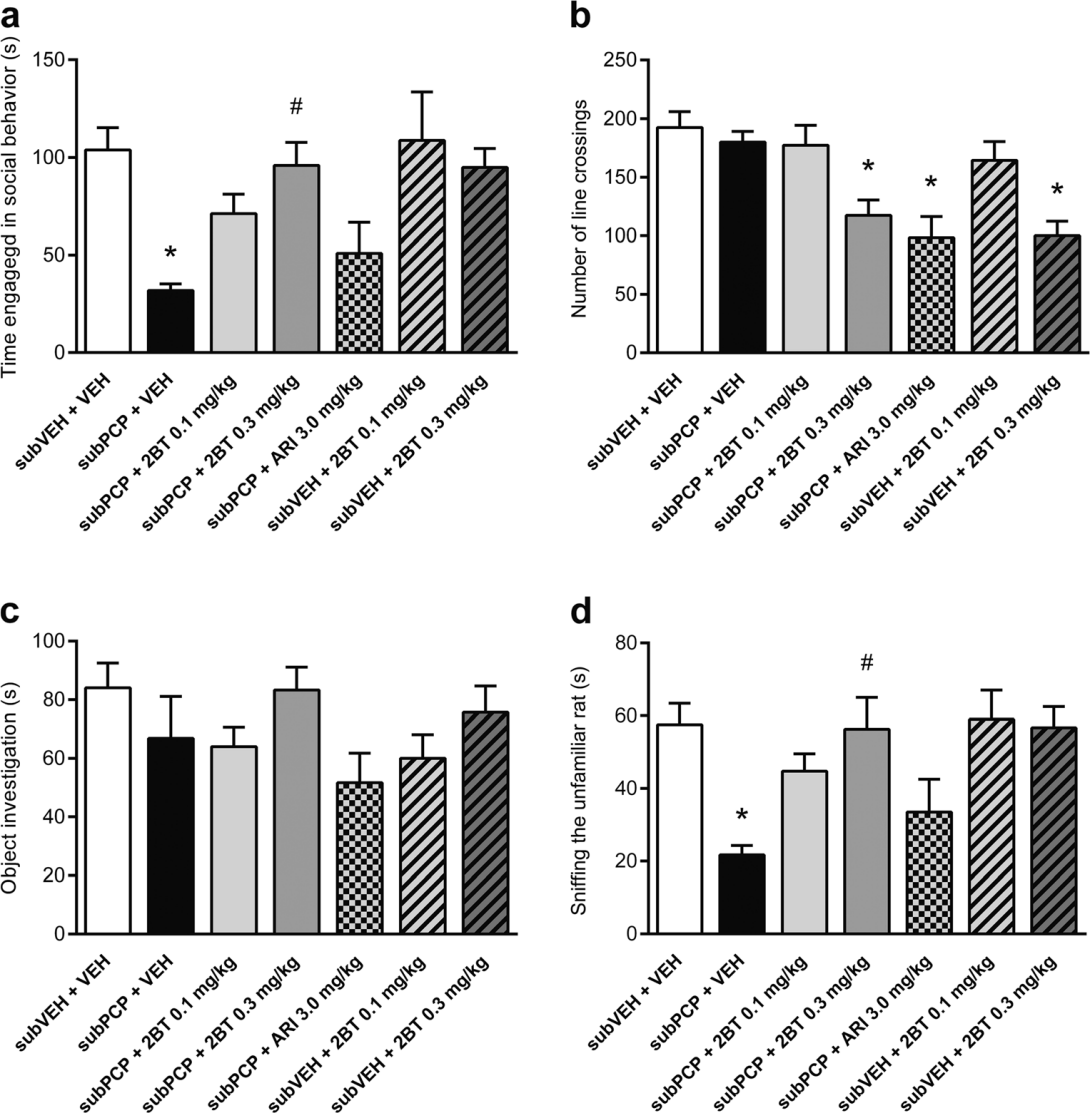
Effects of 2-bromoterguride (0.1 and 0.3 mg/kg) and aripiprazole (3.0 mg/kg) after subchronic phencyclidine (5.0 mg/kg) treatment, and 2-bromoterguride (0.1 and 0.3 mg/kg) alone on the **a** social interaction behavior, **b** total number of line crossings, **c** exploration time of a novel object, and **d** investigative sniffing time towards the unfamiliar rat in the social interaction test in male rats. Data are expressed as mean + SEM of *n* = 7 pairs of unfamiliar rats per group. ^*^ *P <* 0.05 versus controls (subVEH + VEH); ^#^ *P <* 0.05 versus phencyclidine (subPCP + VEH). 2BT: 2-bromoterguride; ARI: aripiprazole; PCP: phencyclidine; VEH: vehicle

Treatment affected our measures of social behavior (*F*_(6,42)_ = 4.4, *P =* 0.001). Subchronic PCP administration induced deficits in social interaction compared to controls (*P =* 0.003), which were ameliorated by 2-bromoterguride (0.3 mg/kg: *P =* 0.009) but not aripiprazole (Fig. 4a)*.* Further, 2-bromoterguride alone did not affect social interaction, implying that the D_2_ receptor partial agonist does not negatively influence sociality in naive rats (Fig. 4a). Drug treatment also affected the mean number of line crossings (distance traveled; *F*_(6,42)_ = 7.8, *P <* 0.001). PCP in combination with aripiprazole and 2-bromoterguride (0.3 mg/kg), or 0.3 mg/kg 2-bromoterguride alone, reduced the number of line crossings compared to controls (*P <* 0.05; Fig. 4b). The average duration investigating a novel object placed in the arena during the social interaction test was not affected by treatments (Fig. 4c). Finally, treatment affected sniffing behavior (*F*_(6,42)_ = 4.6, *P =* 0.001). PCP treatment significantly decreased time sniffing the unfamiliar rat compared to the control group (*P =* 0.011). This effect was ameliorated by 2-bromoterguride (0.3 mg/kg: *P =* 0.014) (Fig. 4d). Climbing and avoiding behavior were unaffected by treatments (data not shown).

### Prolactin

**Fig. 5:**
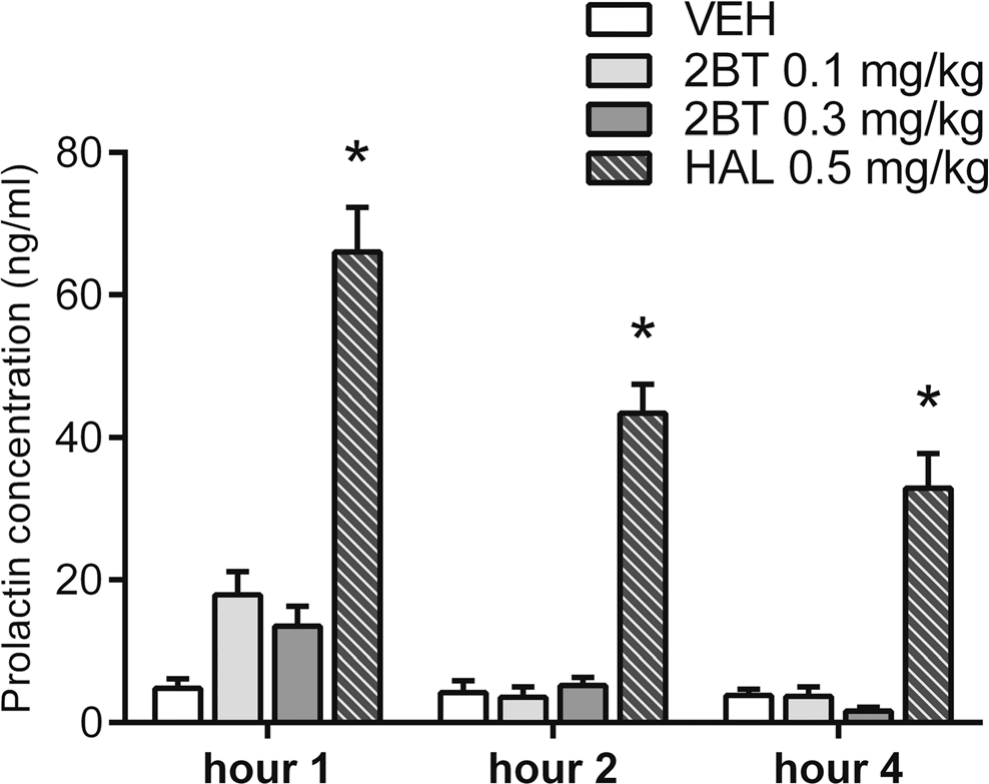
Effects of 2-bromoterguride (0.1 and 0.3 mg/kg) and haloperidol (0.5 mg/kg) on prolactin concentration, 1, 2, or 4 h after administration, in blood serum of male rats. Data are expressed as mean + SEM of *n* = 6–8 rats per group. ^*^ *P <* 0.05 versus controls (VEH)

Treatment affected prolactin concentration in rat blood serum (*F*_(3,37)_ = 78.0, *P <* 0.001). We observed an interaction between the factors treatment and time (*F*_(6,37)_ = 2.9, *P =* 0.021). Administration of haloperidol but not 2-bromoterguride (0.1 and 0.3 mg/kg) or vehicle resulted in elevated prolactin concentrations at all three time points (*P <* 0.001) (Fig. 5).

## Discussion

Achieving cognitive improvement in patients with schizophrenia represents a critical challenge as cognitive impairment diminish the patient’s functional outcome and ability to reintegrate into society (Green et al. 2004; Young and Geyer 2015; Vreeker et al. 2015). Deficits in attention, information processing, and ability to filter out redundant environmental stimuli have been identified in patients with schizophrenia, and PPI has been extensively investigated in patients as well as in preclinical animal models (for review, see Swerdlow et al. 2008). Based on the observation that the startle response magnitude is reduced when a startle eliciting acoustic stimulus is preceded by a weaker acoustic prepulse, the PPI paradigm in rodents represents a preclinical test with face, predictive, and construct validity (Swerdlow et al. 1994; Geyer et al. 2001; Leumann et al. 2002). In this study, the ergoline derivate 2-bromoterguride prevented the apomorphine-induced loss of PPI to the same extent as the dopamine D_2_ antagonist haloperidol, verifying its dopamine D_2_ antagonistic profile and antipsychotic-like effect in rats. PPI disruptions induced by NMDA antagonists seems to be more sensitive to clozapine-like atypical APDs than to typical APDs and hence, the NMDA model of disrupted PPI can aid the identification of novel and atypical antipsychotics (Geyer et al. 2001). Interestingly, 2-bromoterguride also antagonized the PCP-induced loss of PPI to the same extent as clozapine, highlighting the atypical character of 2-bromoterguride. It should be mentioned that the PPI loss induced by NMDA receptor antagonists is mediated by systems other than the central dopamine systems (Keith et al. 1991). The highly potent α_2C_-adrenoceptor antagonist properties of 2-bromoterguride (pA_2_ = 10.5; Jantschak et al. 2013) may contribute to the reversal of PCP-induced impairment of PPI. This is in line with observations using selective α_2C_-adrenoceptor antagonists such as JP-1302 and ORM-10921 as inhibitors of PCP-induced PPI deficits (Sallinen et al. 2007, 2013).

Although the complete etiology of the negative and cognitive symptoms of schizophrenia are not yet understood, the involvement of a dysfunctional glutamatergic system is supported by the observation that NMDA receptor antagonists effectively and reliably produce behavioral and cognitive deficits that mimic features and symptoms of schizophrenia (for reviews, see Neill et al. 2010; Gururajan et al. 2010). The NOR task offers a relative simple method to assess recognition memory as it does not require external motivation or pretraining of animals and relies on the innate explorative behavior of rodents and their preference for novel over familiar objects (Ennaceur and Delacour 1988; for review, see Grayson et al. 2015). Several studies have repeatedly found that NMDA receptor antagonists such as PCP induce memory and learning deficits in animals, and the NOR test is frequently accommodated to assess the effects of novel drugs on PCP-induced deficits (for reviews, see Jentsch and Roth 1999; Meltzer et al. 2013; Neill et al. 2010). It has been suggested that an elevated acetylcholine and dopamine tone in the prefrontal cortex due to 5-HT_1A_ and D_1_ receptor activation may explain why some atypical APDs rescue NOR performance after PCP treatment (Snigdha et al. 2011; Guo et al. 2009; Nagai et al. 2009; for review, see Lyon et al. 2012). Interestingly, 2-bromoterguride shows no affinity for 5-HT_1A_ receptors in vitro (unpublished data). However, the results in this study, showing that 2-bromoterguride attenuates subchronic PCP-induced NOR deficits, indicate that this drug may have effects via D_1_ receptors in addition to its high affinity for 5-HT_2A_ receptors.

Asociality, anhedonia, blunted affect, alogia, and avolition are negative core symptoms of schizophrenia, yet available treatments have only inadequate therapeutic effect (Neill et al. 2014). Negative symptoms as well as cognitive symptoms of schizophrenia impact the patient tremendously. As a consequence, efforts to find reliable research models have promoted the development of various tests for social behaviors in animals (Millan and Bales 2013). In this study, we performed a social interaction test following subchronic PCP treatment in rats to investigate the effect of 2-bromoterguride on social aversion. Our results show that 2-bromoterguride efficiently antagonized the effect of subchronic PCP and restored social behaviors. Surprisingly, in our study, aripiprazole did not ameliorate the social interaction deficits. Line crossings were affected by aripiprazole as well as by 2-bromoterguride; however, sniffing time towards the unfamiliar rat was ameliorated by 2-bromoterguride but not by aripiprazole. A plausible explanation for these differences may be that the 2-bromoterguride-treated rats remained stationary during sniffing bouts towards the unfamiliar rat. As a dopamine D_2_ receptor partial agonist, 2-bromoterguride resembles aripiprazole; however, 2-bromoterguride possesses higher affinity for 5-HT_2A_ receptors than aripiprazole. In addition, 2-bromoterguride is a potent α_2C_-adrenoceptor antagonist (see above). α_2C_-adrenoceptor blockade has been shown to contribute to improvement of cognitive and social function in rats (Marcus et al. 2005; Wadenberg et al. 2007; Sallinen et al. 2013; Uys et al. 2016). These mechanistic properties of 2-bromoterguride may explain why this drug was effective in the social interaction test.

Finally, to extend the side effect profile of 2-bromoterguride, we examined the effect of 2-bromoterguride on prolactin secretion, as dopamine D_2_ receptor blockade may result in elevated levels of secreted prolactin from the anterior pituitary gland (Freeman et al. 2000). It has been hypothesized that elevated prolactin induced by APDs is associated with a cluster of sexual and reproductive complications (for review, see Peuskens et al. 2014). Interestingly, aripiprazole, a D_2_ partial agonist just like 2-bromoterguride, has the potential to improve sexual dysfunction (Hanssens et al. 2008), which is commonly associated with the negative symptoms of schizophrenia (Leucht et al. 2013). Our results presented in this study indicate that 2-bromoterguride does not cause hyperprolactinemia in the acute state of treatment and thus might be associated with less sexual dysfunction than APDs which induce hyperprolactinemia.

In conclusion, our data demonstrate that the dopamine D_2_ receptor partial agonist 2-bromoterguride does not only inhibit amphetamine-induced locomotion (AIL) and conditioned avoidance response (CAR) in rats without inducing catalepsy or causing weight gain (Franke et al. 2016); it also elicits a positive impact on cognitive impairments and social aversion in rats. 2-Bromoterguride prevented the PPI disrupting effects of apomorphine and PCP similar to haloperidol and clozapine, respectively. However, 2-bromoterguride also attenuated object recognition memory deficits, in contrast to clozapine. Moreover, 2-bromoterguride ameliorated subchronic PCP-induced social interaction impairments, an effect that was not shown by aripiprazole. The mechanistic properties and antipsychotic-like effects of 2-bromoterguride previously shown (Jantschak et al. 2013; Franke et al. 2016) and the results presented herein confirm our opinion that 2-bromoterguride represents a very promising third-generation antipsychotic candidate.

## References

[CR1] Aznar S, Hervig MS (2016). The 5-HT_2A_ serotonin receptor in executive function: implications for neuropsychiatric and neurodegenerative diseases. Neurosci Biobehav Rev.

[CR2] Bakshi VP, Swerdlow NR, Geyer MA (1994). Clozapine antagonizes phencyclidine-induced deficits in sensorimotor gating of the startle response. J Pharmacol Exp Ther.

[CR3] Bruins Slot LA, Kleven MS, Newman-Tancredi A (2005). Effects of novel antipsychotics with mixed D_2_ antagonist/5-HT_1A_ agonist properties on PCP-induced social interaction deficits in the rat. Neuropharmacology.

[CR4] de Bartolomeis A, Tomasetti C, Iasevoli F (2015). Update on the mechanism of action of aripiprazole: translational insights into antipsychotic strategies beyond dopamine receptor antagonism. CNS Drugs.

[CR5] Dunlop J, Brandon NJ (2015). Schizophrenia drug discovery and development in an evolving era: are new drug targets fulfilling expectations?. J Psychopharmacol.

[CR6] Ennaceur A (2010). One-trial object recognition in rats and mice: methodological and theoretical issues. Behav Brain Res.

[CR7] Ennaceur A, Delacour J (1988). A new one-trial test for neurobiological studies of memory in rats. 1: behavioral data. Behav Brain Res.

[CR8] Franke RT, Tarland E, Fink H, Pertz HH, Brosda J (2016). 2-Bromoterguride-a potential atypical antipsychotic drug without metabolic effects in rats. Psychopharmacology.

[CR9] Freeman ME, Kanyicska B, Lerant A, Nagy G (2000) Prolactin: structure, function, and regulation of secretion. Physiol Rev 80:1523–1631 10.1152/physrev.2000.80.4.1523 11015620

[CR10] Geyer MA, Krebs-Thomson K, Braff DL, Swerdlow NR (2001). Pharmacological studies of prepulse inhibition models of sensorimotor gating deficits in schizophrenia: a decade in review. Psychopharmacology.

[CR11] Geyer MA (1998). Behavioral studies of hallucinogenic drugs in animals: implications for schizophrenia research. Pharmacopsychiatry.

[CR12] Grayson B, Leger M, Piercy C, Adamson L, Harte M, Neill JC (2015). Assessment of disease-related cognitive impairments using the novel object recognition (NOR) task in rodents. Behav Brain Res.

[CR13] Grayson B, Idris NF, Neill JC (2007). Atypical antipsychotics attenuate a sub-chronic PCP-induced cognitive deficit in the novel object recognition task in the rat. Behav Brain Res.

[CR14] Green MF, Kern RS, Heaton RK (2004). Longitudinal studies of cognition and functional outcome in schizophrenia: implications for MATRICS. Schizophr Res.

[CR15] Guo Y, Zhang H, Chen X, Cai W, Cheng J, Yang Y, Jin G, Zhen X (2009). Evaluation of the antipsychotic effect of bi-acetylated l-stepholidine (l-SPD-A), a novel dopamine and serotonin receptor dual ligand. Schizophr Res.

[CR16] Gururajan A, Taylor DA, Malone DT (2010). Current pharmacological models of social withdrawal in rats: relevance to schizophrenia. Behav Pharmacol.

[CR17] Hanssens L, L'Italien G, Loze JY, Marcus RN, Pans M, Kerselaers W (2008). The effect of antipsychotic medication on sexual function and serum prolactin levels in community-treated schizophrenic patients: results from the Schizophrenia Trial of Aripiprazole (STAR) study (NCT00237913). BMC Psychiatry.

[CR18] Horiguchi M, Hannaway KE, Adelekun AE, Jayathilake K, Meltzer HY (2012). Prevention of the phencyclidine-induced impairment in novel object recognition in female rats by co-administration of lurasidone or tandospirone, a 5-HT_1A_ partial agonist. Neuropsychopharmacology.

[CR19] Jantschak F, Brosda J, Franke RT, Fink H, Möller D, Hübner H, Gmeiner P, Pertz HH (2013). Pharmacological profile of 2-bromoterguride at human dopamine D_2_, porcine serotonin 5-hydroxytryptamine 2A, and α_2C_-adrenergic receptors, and its antipsychotic-like effects in rats. J Pharmacol Exp Ther.

[CR20] Jentsch JD, Roth RH (1999). The neuropsychopharmacology of phencyclidine: from NMDA receptor hypofunction to the dopamine hypothesis of schizophrenia. Neuropsychopharmacology.

[CR21] Jentsch JD, Taylor JR, Roth RH (1998). Subchronic phencyclidine administration increases mesolimbic dopaminergic system responsivity and augments stress- and psychostimulant-induced hyperlocomotion. Neuropsychopharmacology.

[CR22] Keith VA, Mansbach RS, Geyer MA (1991). Failure of haloperidol to block the effects of phencyclidine and dizocilpine on prepulse inhibition of startle. Biol Psychiatry.

[CR23] Kim SF, Huang AS, Snowman AM, Teuscher C, Snyder SH (2007). Antipsychotic drug-induced weight gain mediated by histamine H_1_ receptor-linked activation of hypothalamic AMP-kinase. Proc Natl Acad Sci U S A.

[CR24] Leucht S, Cipriani A, Spineli L, Mavridis D, Orey D, Richter F, Samara M, Barbui C, Engel RR, Geddes JR, Kissling W, Stapf MP, Lässig B, Salanti G, Davis JM (2013). Comparative efficacy and tolerability of 15 antipsychotic drugs in schizophrenia: a multiple-treatments meta-analysis. Lancet.

[CR25] Leumann L, Feldon J, Vollenweider FX, Ludewig K (2002). Effects of typical and atypical antipsychotics on prepulse inhibition and latent inhibition in chronic schizophrenia. Biol Psychiatry.

[CR26] Lyon L, Saksida LM, Bussey TJ (2012). Spontaneous object recognition and its relevance to schizophrenia: a review of findings from pharmacological, genetic, lesion and developmental rodent models. Psychopharmacology.

[CR27] Löschmann PA, Horowski R, Wachtel H (1992) Bromerguride—an ergoline derivative with atypical neuroleptic properties. Clin Neuropharmacol 15(Suppl 1):263A–264A 10.1097/00002826-199201001-00137 1354031

[CR28] Mansbach RS, Geyer MA, Braff DL (1988). Dopaminergic stimulation disrupts sensorimotor gating in the rat. Psychopharmacology.

[CR29] Marcus MM, Jardemark KE, Wadenberg ML, Langlois X, Hertel P, Svensson TH (2005). Combined α_2_ and D_2/3_ receptor blockade enhances cortical glutamatergic transmission and reverses cognitive impairment in the rat. Int J Neuropsychopharmacol.

[CR30] Meltzer HY (2013). Update on typical and atypical antipsychotic drugs. Annu Rev Med.

[CR31] Meltzer HY, Rajagopal L, Huang M, Oyamada Y, Kwon S, Horiguchi M (2013). Translating the N-methyl-D-aspartate receptor antagonist model of schizophrenia to treatments for cognitive impairment in schizophrenia. Int J Neuropsychopharmacol.

[CR32] Millan MJ, Bales KL (2013). Towards improved animal models for evaluating social cognition and its disruption in schizophrenia: the CNTRICS initiative. Neurosci Biobehav Rev.

[CR33] Nagai T, Murai R, Matsui K, Kamei H, Noda Y, Furukawa H, Nabeshima T (2009). Aripiprazole ameliorates phencyclidine-induced impairment of recognition memory through dopamine D_1_ and serotonin 5-HT_1A_ receptors. Psychopharmacology.

[CR34] Neill JC, Harte MK, Haddad PM, Lydall ES, Dwyer DM (2014). Acute and chronic effects of NMDA receptor antagonists in rodents, relevance to negative symptoms of schizophrenia: a translational link to humans. Eur Neuropsychopharmacol.

[CR35] Neill JC, Barnes S, Cook S, Grayson B, Idris NF, McLean SL, Snigdha S, Rajagopal L, Harte MK (2010). Animal models of cognitive dysfunction and negative symptoms of schizophrenia: focus on NMDA receptor antagonism. Pharmacol Ther.

[CR36] Oyamada Y, Horiguchi M, Rajagopal L, Miyauchi M, Meltzer HY (2015) Combined serotonin 5-HT_1A_ agonism, 5-HT_2A_ and dopamine D_2_ receptor antagonism reproduces atypical antipsychotic drug effects on phencyclidine-impaired novel object recognition in rats. Behav Brain Res 285:165–175 10.1016/j.bbr.2014.09.040 25448429

[CR37] Peuskens J, Pani L, Detraux J, De Hert M (2014). The effects of novel and newly approved antipsychotics on serum prolactin levels: a comprehensive review. CNS Drugs.

[CR38] Sallinen J, Höglund I, Engström M, Lehtimäki J, Virtanen R, Sirviö J, Wurster S, Savola JM, Haapalinna A (2007). Pharmacological characterization and CNS effects of a novel highly selective α_2C_-adrenoceptor antagonist JP-1302. Br J Pharmacol.

[CR39] Sallinen J, Holappa J, Koivisto A, Kuokkanen K, Chapman H, Lehtimäki J, Piepponen P, Mijatovic J, Tanila H, Virtanen R, Sirviö J, Haapalinna A (2013). Pharmacological characterisation of a structurally novel α_2C_-adrenoceptor antagonist ORM-10921 and its effects in neuropsychiatric models. Basic Clin Pharmacol Toxicol.

[CR40] Snigdha S, Idris N, Grayson B, Shahid M, Neill JC (2011). Asenapine improves phencyclidine-induced object recognition deficits in the rat: evidence for engagement of a dopamine D_1_ receptor mechanism. Psychopharmacology.

[CR41] Snigdha S, Neill JC (2008). Efficacy of antipsychotics to reverse phencyclidine-induced social interaction deficits in female rats—a preliminary investigation. Behav Brain Res.

[CR42] Snigdha S, Neill JC (2008). Improvement of phencyclidine-induced social behaviour deficits in rats: involvement of 5-HT_1A_ receptors. Behav Brain Res.

[CR43] Swerdlow NR, Weber M, Qu Y, Light GA, Braff DL (2008). Realistic expectations of prepulse inhibition in translational models for schizophrenia research. Psychopharmacology.

[CR44] Swerdlow NR, Bakshi V, Geyer MA (1996). Seroquel restores sensorimotor gating in phencyclidine-treated rats. J Pharmacol Exp Ther.

[CR45] Swerdlow NR, Braff DL, Taaid N, Geyer MA (1994). Assessing the validity of an animal model of deficient sensorimotor gating in schizophrenic patients. Arch Gen Psychiatry.

[CR46] Ücok A, Gaebel W (2008). Side effects of atypical antipsychotics: a brief overview. World Psychiatry.

[CR47] Uys M, Shahid M, Sallinen J, Dreyer W, Cockeran M, Harvey BH (2016). The α_2C_-adrenoceptor antagonist, ORM-10921, has antipsychotic-like effects in social isolation reared rats and bolsters the response to haloperidol. Prog Neuro-Psychopharmacol Biol Psychiatry.

[CR48] Vreeker A, van Bergen AH, Kahn RS (2015). Cognitive enhancing agents in schizophrenia and bipolar disorder. Eur Neuropsychopharmacol.

[CR49] Wadenberg ML, Wiker C, Svensson TH (2007). Enhanced efficacy of both typical and atypical antipsychotic drugs by adjunctive α_2_ adrenoceptor blockade: experimental evidence. Int J Neuropsychopharmacol.

[CR50] Wilson CA, Koenig JI (2014). Social interaction and social withdrawal in rodents as readouts for investigating the negative symptoms of schizophrenia. Eur Neuropsychopharmacol.

[CR51] Young JW, Geyer MA (2015). Developing treatments for cognitive deficits in schizophrenia: the challenge of translation. J Psychopharmacol.

